# Hospital Meetings, &c.

**Published:** 1897-05-29

**Authors:** 


### HOSPITAL MEETINGS, &C.

### BRITISH HOME FOR INCURABLES.

The annual meeting of the British Home for Incurables was held at the City Terminus Hotel on Tuesday, 18th inst., Lord Amherst in the chair. Five in-patients and 15 pensioners were elected.

### THE HOSPITAL FOR EPILEPSY AND PARALYSIS.

The Bishop of Stepney, who occupied the chair at the annual meeting of the Hospital for Epilepsy, Regent's Park, N.W., on Wednesday, the 19th inst., emphasised the fact that in three years the lease of the building at present occupied would expire, and strongly urged upon the committee the need of a new building, which, according to the annual report, would cost from £10,000 to £15,000. He hoped they would use a difficulty, which must soon assume a critical form, as a stepping-stone to a higher success. At the same time he felt that the present year, in which funds of all kinds and expenditure unusual in both character and extent were becoming almost universal, was one in which it would be specially difficult, if not impossible, to commence such a fund. The report and accounts were adopted, and the three retiring members of the Managing Committee were re-elected. Mr. Pearce Gould, hon. surgeon to the hospital, reminded the meeting that it was at this hospital that the first operation for the removal of a brain tumour had been performed on the living subject, and asserted that a modern building was a necessity, from the point of view of the staff, no less than in the interests of the patients.

### PADDINGTON GREEN CHILDREN'S HOSPITAL.

From the report of the Paddington Green Children's Hospital, which was adopted at the annual meeting held at the hospital on Wednesday, the 19th inst., we find that during 1896 481 in and 10,407 out patients, as well as 2,117 casualties and accidents, received treatment. The cost of maintaining the hospital was £2,887, while the ordinary income amounted to £2,846, thus showing a small deficiency. Legacies were received amounting to £1,123, and Mr. George Woodhouse gave a donation of £1,000 for the perpetual endowment of a cot. £1,769 was received on account of the building fund, and £3,319 was expended under this head. The committee drew attention to the scanty support given to the convalescent home, the total income having been only £102, against an expenditure of £512, leaving a deficiency of over £400 to be provided out of the hospital funds. They fear, unless more liberal aid is forthcoming, that they will be obliged to give up this most useful adjunct to the work of the hospital, to which seventy-six little children were sent during 1896.

### SAMARITAN FREE HOSPITAL.

This being the fiftieth year of the Samaritan Free Hospital's existence, a festival dinner in aid of its funds was held at the Whitehall Rooms on Friday, 21st inst. Lord Leigh, the president of the hospital, took the chair in the absence of the Duke of Connaught, who had been commanded by the Queen to attend Her Majesty on the occasion of her visit to Sheffield. In a letter to the committee, however, the Duke of Connaught expressed his wish that every success might attend the festival. Among those present were the Hon. Miss Leigh, the Portuguese Minister, Sir J. H. Johnson, General Montague, General Blake, Colonel and Mrs. Gordon Watson, Sir George Bowen, and Sir Horace Farquhar, M.P.

The Chairman, in proposing the toast of the evening—"Prosperity to the Samaritan Free Hospital"—alluded to the death of Sir Spencer Wells, to whom the success which had attended the treatment of ovarian cases at the Samaritan Free Hospital had been so largely due. During the past year no less a sum than £400 had been contributed by the patients as thank-offerings for the relief they had received. In 1896 the in-patients treated had numbered 588 and the out-patients had amounted to nearly 10,000—a very large increase on former years. Mr. Edward Nash, chairman of the committee of management, responded. Contributions amounting to £1,748 were announced during the evening.

### NATIONAL REFUGES FOR HOMELESS AND DESTITUTE CHILDREN.

The EARL OF JERSEY presided over the fifty-fourth annual meeting of this institution which was held at Exeter Hall on Tuesday, 25th inst. The large hall in which the meeting was held was filled to overflowing, and the platform was occupied by a choir of some five hundred children, including the boys of the training ships *Arethusa* and *Chichester* and the girls from the society's schools at Sudbury and Ealing. The report (which was adopted on the motion of the Chairman, seconded by the Rev. A. J. Poynder, vicar of St. Michael's, Burleigh Street, Strand) stated that the total number of children admitted to the refuges up to the end of 1896 was 14,236, viz., 11,892 boys and 2,344 girls. The receipts for the year 1896 amounted to £23,979, and the payments to £22,026.—The Secretary in reading the report said that out of every shilling subscribed 10½d. went to the feeding and clothing of the children.—A resolution, asserting that the objects contemplated by the National Refuges, viz., "the rescuing of orphan and neglected children and the giving them a sound Christian education, combined with industrial training, are eminently calculated to diminish vagrancy and crime, and to raise such children to become honourable members of society," was carried unanimously on the motion of the Dean of Norwich, seconded by the Rev. R. Calder Gillie.—The mover, in the course of an eloquent address, said that of all the characteristics of this Victorian era that characteristic which was most full of hope and of enthusiastic appeal to the gratitude of mankind was the fact that this age might be called the age of the child.—During the evening choruses were rendered by the choir, the boys of the Farm School at Bisley gave a musical drill display, and a ten minutes' illustration of the work of the society was given by means of a lantern exhibition. A telegram of congratulation on the completion of the sixtieth year of her reign was sent to Her Majesty the Queen on behalf of the meeting.

### OTHER MEETINGS.

During the past week the following hospitals have held their annual meetings: The Victoria Hospital for Children, on the 19th inst.; the Hospital for Women, on the 20th inst.; and the Alexandra Hospital for Children, on the 24th inst.

